# Protective role of 5-azacytidine on myocardial infarction is associated with modulation of macrophage phenotype and inhibition of fibrosis

**DOI:** 10.1111/jcmm.12248

**Published:** 2014-02-27

**Authors:** Yong Sook Kim, Wan Seok Kang, Jin Sook Kwon, Moon Hwa Hong, Hye-yun Jeong, Hae Chang Jeong, Myung Ho Jeong, Youngkeun Ahn

**Affiliations:** a Heart Research Center, Chonnam National University HospitalGwangju, South Korea; b Research Laboratory of Cardiovascular Regeneration, Chonnam National University HospitalGwangju, South Korea; c Center for Molecular Medicine, Graduate School, Chonnam National UniversityGwangju, South Korea; d Department of Cardiology, Chonnam National University HospitalGwangju, South Korea

**Keywords:** 5-azacytidine, macrophage, inducible nitric oxide synthase, myocardial infarction

## Abstract

We examined whether a shift in macrophage phenotype could be therapeutic for myocardial infarction (MI). The mouse macrophage cell line RAW264.7 was stimulated with peptidoglycan (PGN), with or without 5-azacytidine (5AZ) treatment. MI was induced by ligation of the left anterior descending coronary artery in rats, and the rats were divided into two groups; a saline-injection group and a 5AZ-injection group (2.5 mg/kg/day, intraperitoneal injection). LV function was evaluated and immunohistochemical analyses were performed 2 weeks after MI. Cardiac fibrosis was induced by angiotensin II (AngII) infusion with or without 5AZ (5 mg/kg/day) in mice. Nitric oxide was produced by PGN, which was reduced by 77.87% after 5AZ treatment. Both induction of inducible nitric oxide synthase (iNOS) and iNOS promoter activity by PGN were inhibited by 5AZ. Ejection fraction (59.00 ± 8.03% *versus* 42.52 ± 2.58%), contractility (LV dP/dt-max, 8299.76 ± 411.56 mmHg *versus* 6610.36 ± 282.37 mmHg) and relaxation indices (LV dP/dt-min, −4661.37 ± 210.73 mmHg *versus* −4219.50 ± 162.98 mmHg) were improved after 5AZ administration. Cardiac fibrosis in the MI+5AZ was 8.14 ± 1.00%, compared with 14.93 ± 2.98% in the MI group (*P* < 0.05). Arginase-1(+)CD68(+) macrophages with anti-inflammatory phenotype were predominant in the infarct border zone of the MI+5AZ group, in comparison with the MI group. AngII-induced cardiac fibrosis was also attenuated after 5AZ administration. In cardiac fibroblasts, pro-fibrotic mediators and cell proliferation were increased by AngII, and these increases were attenuated after 5AZ treatment. 5AZ exerts its cardiac protective role through modulation of macrophages and cardiac fibroblasts.

## Introduction

The wound healing of the infarcted myocardium is a dynamic process that comprises a series of events culminating in the removal of the injured tissue and the establishment of a scar [1].

Monocytes are a major source of both pro- and anti-inflammatory cytokines. Under physiological conditions, monocytes circulate in the bloodstream in a quiescent state, but can be quickly activated. Once activated, monocytes differentiate into macrophages, are recruited to the sites of injury and play a pivotal role in tissue remodelling and healing.

Macrophages undergo either classic activation (M1, pro-inflammatory type) or alternative activation (M2, anti-inflammatory type) in response to various signals, and M1 and M2 macrophages have distinct functions [2]. M1 macrophages mediate defence of the host from various pathogens. M2 macrophages scavenge debris, promote tissue repair and regulate immune functions. Inducible nitric oxide synthase (iNOS), cyclooxygenase-2 (Cox-2) and tumour necrosis factor-α (TNF-α) are associated with M1 macrophages, and arginase-1 (Arg1), CD206 (mannose receptor) and resistin-like α (Fizz1) are associated with M2 macrophages. Infiltrated macrophages are intimately involved in cardiac damage and repair in the myocardium. Macrophage infiltration into the infarcted myocardium regulates multiple wound healing functions, including phagocytosis and wound debridement, angiogenesis, fibroblast activation and proliferation, and collagen metabolism [3–5].

Nitric oxide participates both in the normal function and in the pathology of ischaemic injury of the heart. Inducible nitric oxide synthase is an important enzyme implicated in the classic activation of macrophages. Various extracellular stresses activate iNOS pathways in immune cells. Cytokines released from damaged host cells induce iNOS expression to produce nitric oxide. This process includes the degradation of inhibitor of nuclear factor kappa B (IκB), which releases the transcription factor nuclear factor kappa B (NF-κB). Nuclear factor kappa B is translocated from the cytosol to the nucleus, where it binds to κB elements in the iNOS 5′ flanking region, triggering iNOS transcription [6]. Nuclear factor kappa B is a well-known key transcription factor that regulates iNOS expression. In the resting state, NF-κB is bound to IκB to retain NF-κB in the cytosol.

Peptidoglycan (PGN), the major component of the cell wall of Gram-positive bacteria, is a representative toll-like receptor 2 (TLR2) ligand and activates the innate immune system to release cytokines and chemokines. Toll-like receptor 2 was reported to be induced in infarcted myocardium, and TLR2 activation has a critical role in cardiac remodelling [7].

5-azacytidine (5AZ) is an inhibitor of DNA methylation and is a curative epigenetic drug for myelodysplastic syndromes. 5-azacytidine has been utilized to induce myogenic transdifferentiation [8] and to partially differentiate marrow stromal cells into cardiomyocytes [9,10]. Furthermore, systemic administration of 5AZ blocks renal fibrosis by selective targeting of renal fibroblasts [11].

Here, we elucidate whether the phenotype of polarized M1-M2 macrophages can be revised after 5AZ treatment to improve myocardial performance after MI. We show that an anti-inflammatory shift in macrophages and inhibition of fibrosis are involved in 5AZ-mediated cardioprotection.

## Materials and methods

### Cell culture and stimulation

The RAW264.7 murine monocyte/macrophage cell line was purchased from Korean Cell Line Bank (Seoul, Korea) and was cultured in DMEM (Invitrogen, Carlsbad, CA, USA) supplemented with 10% heat-inactivated foetal bovine serum (FBS). RAW264.7 cells were treated with PGN (10 μg/ml; Sigma-Aldrich, St Louis, MO, USA) with or without 5AZ (10 μM; Sigma-Aldrich). BAY11-7082, an inhibitor of NF-κB, was purchased from Sigma-Aldrich.

Bone marrow mononuclear cells (BMMNC) were collected by flushing the femurs and tibias from mice. To differentiate to bone marrow-derived macrophages (BMDM), BMMNC were cultured in RPMI1640 (Invitrogen) containing macrophage colony-stimulating factor (M-CSF, 100 ng/ml; R&D Systems, Minneapolis, MN, USA) for 8 days.

### Measurement of nitrite formation

RAW264.7 cells were seeded in 24-well culture plate and stimulated with PGN with or without 5AZ to measure the amount of nitrite formation. Culture supernatants were collected by centrifugation (10,000 × *g* for 5 min.) and were assayed for nitrite formation by use of Griess reagent according to the manufacturer's instructions (Promega, Madison, WI, USA).

### RNA isolation and RT-PCR

To compare mRNA expression levels, cells were harvested and homogenized in Trizol solution (Invitrogen) according to the manufacturer's instructions. cDNA was synthesized to perform RT-PCR. Glyceraldehyde 3-phosphate dehydrogenase (GAPDH) was used as a loading control. The primers used were as follows: mouse iNOS, forward, 5′-TCACCTTCGAGGGCAGCCGA, and reverse, 5′-TCCGTGGCAAAGCGAGCCAG; mouse Arg1, forward, 5′-CCAGCATTCACCCCGGCGAC, and reverse, 5′-GCCCTTGGGAGGAGAAGGCGT; mouse COX-2, forward, 5′-TTTGTTGAGTCATTCACCAGACAGAT, and reverse, 5′-CAGTATTGAGGAGAACAGATGGGATT; and mouse GAPDH, forward, 5′-TGTGATGGGTGTGAACCACG, and reverse, 5′-CAGTGAGCTTCCCGTTCAGC; mouse interleukin 10 (IL-10) forward, 5′-ACTGGCATGAGGATCAGCAG, and reverse, 5′-CTCCTTGAT TTCTGGGCCAT; mouse CD206 forward, 5′-CTGCAGATGGGTGGGTTATT, and reverse, 5′-GGCATTGATGCTGCTGTTATG; mouse interleukin 4 receptor (IL-4R) forward, 5′-CTAGCTCCGTGCCCTTATTTAC, and reverse, 5′-GGTTGGCTTCTGGTGGTATT; mouse FIZZ1 forward, 5′-CCAATCCAGCTAACTATCCCTCC, and reverse, 5′-ACCCAGTAGCAGTCATCCCA; rat connective tissue growth factor, forward, 5′-ACCGACCTCCTCCAGACGGC, and reverse, 5′-CGTCCAGCACCAGGCTCACG; rat collagen type I (ColI), forward, 5′-CTCCTGACGCATGGCCAAGA, and reverse, 5′-TGGGTCCCTCGACTCCTATG; rat GAPDH, forward, 5′-GGCCAAGGTCATCCATGA, reverse, 5′-TCAGTGAGCCCAGGATG.

### Western blot analysis

Cells were washed with ice-cold PBS, resuspended in lysis buffer (20 mM Tris-HCl, pH7.4, 0.1 mM ethylenediaminetetraacetic acid (EDTA), 150 mM NaCl, 1 mM phenylmethylsulfonyl fluoride, 1 mg/ml leupeptin, 1 mM Na_3_VO_4_) and sonicated briefly. After centrifugation at 10,000 × *g* force for 10 min., the supernatant was prepared as a protein extract. Equal concentrations of proteins were fractionated by electrophoresis on 8% or 10% acrylamide gels and were transferred onto a polyvinylidene fluoride membrane (Millipore, Billerica, MA, USA) followed by blotting with antibodies against iNOS (Abcam, Cambridge, MA, USA), endothelia NOS (eNOS) (Cell Signaling, Danvers, MA, USA), IκBα (Santa Cruz Biotechnology, Dallas, TX, USA), p65 (Santa Cruz), PCNA (Santa Cruz), phosphorylated extracellular signal-regulated kinase (ERK; Cell Signaling), ERK (Cell Signaling), phosphorylated Akt (Cell Signaling), Akt (Cell Signaling), bcl-2 (Santa Cruz) and β-actin (Sigma-Aldrich). Protein levels were determined by using Western Breeze reagents (Santa Cruz) and Image Reader (LAS-3000 Imaging System; Fuji Photo Film, Tokyo, Japan).

### Promoter study

RAW264.7 cells were transfected with iNOS promoter-luciferase (kindly gifted by Professor Myung-Jun Kim, College of Medicine, Catholic University, Seoul, Korea) in triplicate by using Effectene (Qiagen, Valencia, CA, USA) according to the manufacturer's protocol. Twenty-four hours after transfection, cells were washed with PBS, and treated with PGN with or without 5AZ. Cells were harvested and luciferase activity was assayed by using the Dual Luciferase Reporter assay system with normalization to Renilla luciferase activity (Promega).

### Rat model of myocardial infarction

To investigate the role of 5AZ in myocardial infarction, myocardial infarction was induced by permanent ligation of the left anterior descending coronary artery. The study was reviewed and approved by the Chonnam National University Institutional Animal Care and Use Committee (CNU IACUC-H-2010-12). Male Sprague-Dawley rats (weighing 200–230 g) were purchased from Jung Ang Animals (Seoul, Korea). For MI induction, rats were anaesthetized with an intramuscular injection of ketamine (50 mg/kg) and xylazine (5 mg/kg), intubated and mechanically ventilated. The proximal left anterior descending coronary artery was ligated. Finally, the heart was repositioned in the chest, and the chest was closed. The animals remained in a supervised setting until becoming fully conscious. After 1 day of MI, rats were administered with saline (*n* = 6) or 5AZ (2.5 mg/kg of bw in saline, *n* = 6) every other day *via* intraperitoneal injection for 2 weeks.

### Mouse model of cardiac fibrosis

To examine the role of 5AZ on cardiac fibrosis, 8-week-old BALB/c mice were purchased from Jung Ang Animals. The procedure was reviewed and approved by the Chonnam National University Institutional Animal Care and Use Committee (CNU IACUC-H-2010-12). The mice were housed in the animal care facility of Chonnam National University Medical School and had access to food and water *ad libitum*. Mice were anaesthetized by an intramuscular injection of ketamine and xylazine and were divided into four groups (*n* = 8 in each group). Mice were infused with vehicle (saline) or Ang II at a rate of 65 mg/kg of bw per day by use of osmotic minipumps (ALZET model 1002; Cupertino, CA, USA) subcutaneously for 2 weeks. After 24 hrs, 5AZ (5 mg/kg of bw/day in saline) or saline was administered *via* intraperitoneal injection every other day. Two weeks later, systolic blood pressure was measured by right carotid artery cannulation with a Millar catheter (Millar Instruments, Houston, TX, USA).

Then, mice were weighed and then killed *via* cervical dislocation. The chest cavity was rapidly opened, and the heart removed and rinsed in cold saline. Fatty debris and connective tissue were removed, the heart blotted dry, weighed and the heart weight/bw ratio calculated.

### Isolation of cardiac fibroblasts

Primary cardiac fibroblasts were isolated from 2-day-old Sprague-Dawley rat pups. Briefly, hearts were removed, and the ventricles were washed in cold PBS, chopped and digested with 0.1% collagenase type 2 (210 U/ml; Sigma-Aldrich) and pancreatin (0.6 mg/ml; Gibco, Grand Island, NY, USA) for 30 min. with mild stirring. The supernatants were collected and subjected to centrifugation through Percoll gradient (Sigma-Aldrich). The myocytes layer was collected and cultured on a flask in medium supplemented with 10% of FBS. After 1 hr of incubation, non-adherent cells were removed and adherent cells were cultivated. Upon reaching confluence, cells were detached with trypsin-EDTA, split in a 1:3 ratio and cultured.

### LV function measurement

LV function was assessed by echocardiography and haemodynamic changes by Millar catheter. After 2 weeks, the animals were anaesthetized, intubated and mechanically ventilated to study haemodynamic variables with a 1.4 F micromanometer-tipped catheter (Millar Instruments). The catheter was inserted into the right carotid artery and advanced into the left ventricle (LV) to measure pressures, which were analysed with the Chart V5 analysis program (Millar Instruments). Echocardiography was performed to measure LV function. Echocardiographic studies were performed with a 15-MHz linear array transducer system (iE33 system; Philips Medical Systems, Andover, MA, USA) by an expert who was not aware of experimental conditions to exclude bias. Two-dimensional guided M-mode of the LV was obtained from the parasternal view. Left ventricle cavity dimension was measured, and percentage change in LV dimension [fractional shortening (FS), LV%FS] was calculated as: LV%FS = [(LVEDD − LVESD)/LVEDE] × 100, where LVEDD is LV end-diastolic diameter and LVESD is LV end-systolic diameter. LV% ejection fraction (EF) was calculated as: LV%EF = [(EDV − ESV)/EDV] × 100, where EDV is LV volume at end-diastole and ESV is LV volume at end-systole. Left ventricle volume was estimated by the area–length method.

### Immunohistochemical staining

After 24 hrs of MI induction, the heart was excised, 2 mm-thick slices were cut and stained in 1% TTC solution for 30 min. Following staining, the slices were fixed in formalin for 30 min. Infarcted areas were visible as pale, while viable areas were stained red. Slices were scanned for evaluation of infarct size with NIS-Elements Advanced Research program (Nikon, Tokyo, Japan).

At the end of the experiment, the rats were killed, and the hearts were rapidly removed, fixed in formalin and embedded in paraffin for histological studies. For immunohistochemical analysis, slides were treated with 3% hydrogen peroxide in PBS for 10 min. at room temperature to block endogenous peroxidase activity. After non-specific binding was blocked with 5% normal goat serum (Sigma-Aldrich), the slides were incubated with primary antibodies against CD68 (1:100; BMA Biomedicals, Augst, Switzerland), iNOS (Abcam), Arg-1 (1:100; Abcam) or α-smooth muscle actin (Santa Cruz) for 18 hrs at 4°C. Sections were washed with PBS three times and then incubated for 1 hr with Alexa-Fluor 488 or 594 secondary antibodies. After washing, the slides were coverslipped with mounting medium (VectaMount mounting medium; Vector Labs Inc., Burlingame, CA, USA). Images were obtained and digitized on a computer by using an Olympus CX31 microscope (Olympus, Tokyo, Japan) equipped with an Infinity 1 camera (Lumenera Scientific, Ottawa, ON, Canada). Immunofluorescence was detected by using a Carl Zeiss confocal microscope (Carl Zeiss Microscopy, Jena, Germany). Images were obtained by using Zeiss LSM version 3.2 SP2 software. Cardiac fibrosis was measured by Masson's Trichrome staining, and fibrotic areas were measured by visualizing blue-stained fibrotic deposits by using NIS-Elements Advanced Research program (Nikon). The percentage of ventricular fibrosis was calculated as the blue-stained area divided by total ventricular area.

### Statistical analysis

All experiments were performed a minimum of three times. The data are presented as means ± SD. Differences were analysed by Student's *t*-test or one-way anova, followed by Tukey post-hoc test. P-values less than 0.05 were considered significant.

## Results

### Inhibitory effect of 5AZ on PGN-induced nitric oxide generation and iNOS expression in RAW264.7 cells

**Figure 1:**
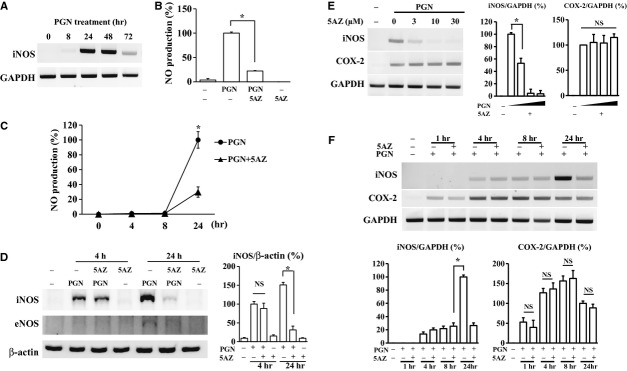
Inhibition by 5-azacytidine (5AZ) of nitric oxide and nitric oxide synthase (iNOS) induced by peptidoglycan (PGN) in RAW264.7 macrophages. (A) iNOS mRNA expression was examined after PGN treatment for indicated times. (B) Nitric oxide produced by PGN (10 μg/ml) was quantified with or without 5AZ (10 μM) treatment. Nitric oxide was generated by PGN stimulation for 24 hrs, which was attenuated by 5AZ in RAW264.7 cells. (C) Nitrite formation was quantified at the indicated time. (D) The protein level of iNOS was increased by PGN and decreased by 5AZ at 24 hrs. (E) PGN-induced expression of iNOS mRNA was inhibited by 5AZ in a dose-dependent manner. Note that mRNA of cyclooxygenase-2 (COX-2) was not affected by 5AZ in the same condition. The mRNA level of iNOS and COX-2 is expressed as graphs. (F) The inhibitory effect of 5AZ (10 μM) on PGN-induced iNOS mRNA was observed at 24 hrs of stimulation. Note that COX-2 mRNA was not affected by 5AZ. The mRNA level of iNOS and COX-2 is expressed as graphs. Results are expressed as mean ± SD. *P < 0.05 compared with the basal level (PGN alone).

Inducible nitric oxide synthase was used as a marker of macrophages with the M1 phenotype. Inducible nitric oxide synthase mRNA expression reached its peak at 24 hrs after PGN treatment (Fig.1A). RAW264.7 macrophages were stimulated with PGN (10 μg/ml) with or without 5AZ (10 μM) for 24 hrs and culture media were collected to measure nitrite formation. 5-azacytidine treatment attenuated PGN-induced nitric oxide production to 22.13% (Fig.1B). In cells treated with PGN for various time intervals, it was found that the inhibition of nitrite formation by 5AZ was apparent between 8 and 24 hrs after PGN stimulation (Fig.1C). This finding indicated that 5AZ might not be involved in a rapid reaction such as NF-κB in response to PGN. The inhibitory effect of 5AZ on PGN-induced iNOS expression was also apparent at 24 hrs after PGN stimulation (Fig.1D). To determine which enzyme was responsible for nitrite formation in response to PGN, expression of iNOS and eNOS was determined by Western blot. eNOS was not induced by PGN in RAW264.7 macrophages, whereas iNOS was shown to be the principal enzyme for nitrite formation in RAW264.7 cells (Fig.1D).

In PGN-stimulated RAW264.7 cells, 5AZ inhibited iNOS expression in a concentration-dependent manner. On the other hand, 5AZ did not influence COX-2 induction by PGN at all (Fig.1E). Inducible nitric oxide synthase and COX-2 are well-known NF-κB targets. Thus, we next examined the effect of 5AZ on iNOS and COX-2 at various time-points. Inducible nitric oxide synthase mRNA was induced by PGN at 4 hrs and reached a maximum level at 24 hrs. 5-azacytidine inhibited PGN-induced iNOS mRNA expression at 24 hrs with statistical significance. On the other hand, COX-2 mRNA was induced by PGN without any interference by 5AZ (Fig.1F). Thus, these results suggested that the inhibitory mechanism of 5AZ on these molecules was not the same.

### Effect of 5AZ on PGN-stimulated bone marrow macrophages

**Figure 2:**
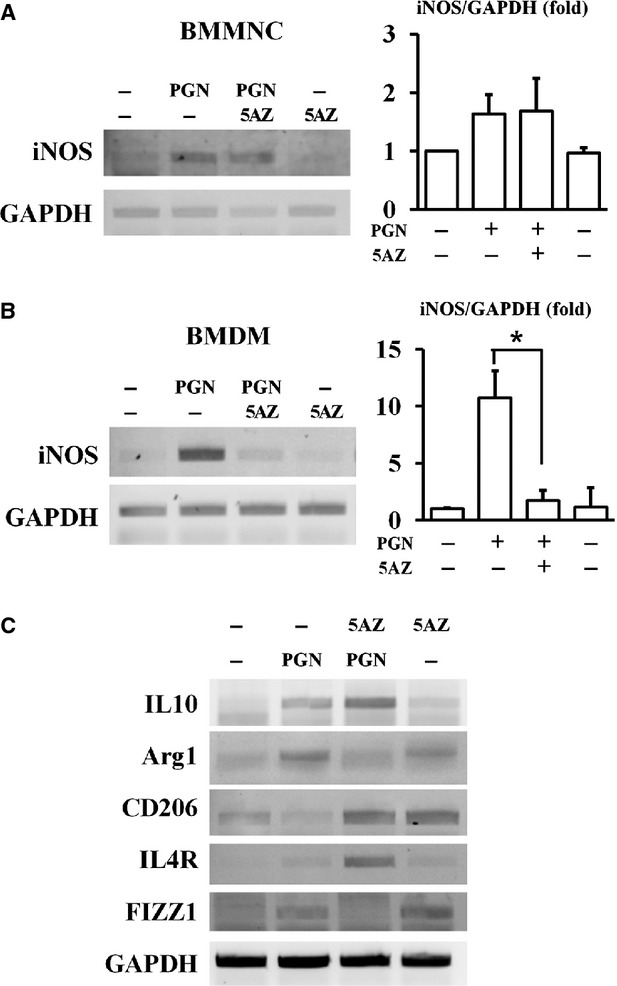
Effects of 5AZ on PGN-induced iNOS mRNA in bone marrow mononuclear cells (BMMNC) and bone marrow-derived macrophages (BMDM). (A) 5AZ did not affect iNOS induction by PGN treatment in BMMNC. (B) In BMDM, PGN-induced iNOS expression was significantly reduced by 5AZ treatment. (C) In RAW264.7 cells, 5AZ affected mRNA levels of anti-inflammatory macrophage markers such as interleukin 10 (IL-10), arginase 1 (Arg1), CD206, interleukin 4 receptor (IL4R) and FIZZ1.

To examine whether the inhibitory effect of 5AZ on iNOS was specific to macrophages, we studied the effect of 5AZ in BMMNC as well as in BMDM. In PGN-stimulated BMMNC, 5AZ did not reduce iNOS mRNA induction (Fig.2A). On the other hand, 5AZ showed a significant inhibition effect on PGN-induced iNOS mRNA in BMDM (Fig.2B).

Next, we examined the effect of 5AZ on anti-inflammatory M2 phenotype markers in addition to iNOS, a pro-inflammatory M1 marker. IL-10, CD206 and IL-4R were induced either by 5AZ co-treatment or 5AZ alone in PGN-stimulated RAW264.7 cells. On the other hand, Arg1 and FIZZ1 were increased by PGN stimulation (Fig.2C).

### Effect of 5AZ on NF-κB activation and iNOS promoter activity in PGN-stimulated RAW264.7 macrophages

**Figure 3:**
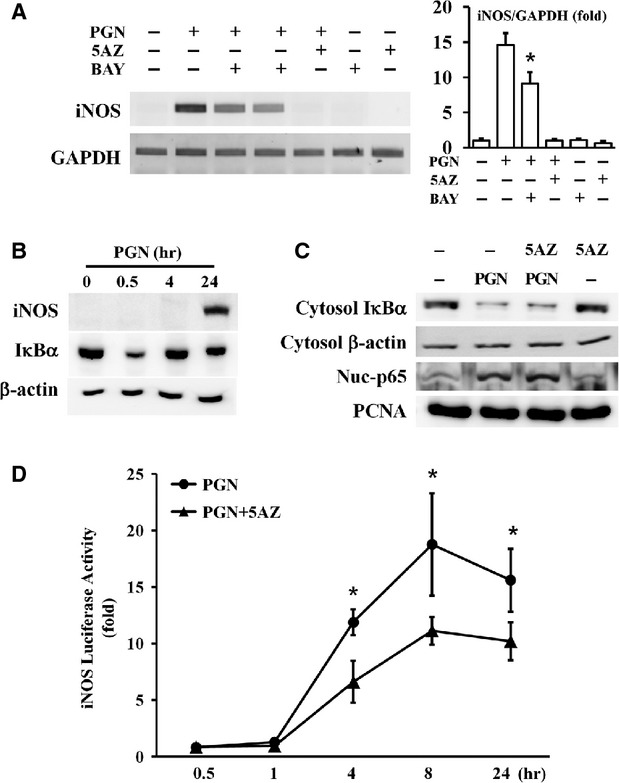
Effect of 5AZ on nuclear factor kappa B (NF-κB) activation and iNOS promoter activity. (A) RAW264.7 cells were stimulated with PGN in the presence of BAY11-7082, an inhibitor of NF-κB. (B) RAW264.7 cells were stimulated with PGN for the indicated time and IκBα was degraded at 30 min. (C) Cells were treated with PGN for 30 min. and cytosolic IκBα was degraded and p65 was translocated to the nuclear fraction. 5AZ did not change PGN-induced IκBα degradation and p65 nuclear translocation. (D) iNOS promoter activity was measured at various times. Promoter activity was significantly increased at 4 hrs and reached a maximum at 8 hrs. Increased promoter activity was reduced after 5AZ treatment.

Because iNOS is a well-known target of NF-κB, the NF-κB response to PGN was examined. In PGN-induced RAW264.7 cells, BAY11-7082, an inhibitor of NF-κB, inhibited iNOS by 37.65%, while 5AZ inhibited iNOS by 93.29% (Fig.3A). To confirm the effect of 5AZ on PGN-activated NF-κB, RAW264.7 cells were stimulated with PGN for 30 min., 4 hrs or 24 hrs. IκBα was degraded at 30 min. of PGN stimulation, and iNOS protein was increased significantly at 24 hrs (Fig.3B). This result suggested that the PGN-induced iNOS expression was mediated by NF-κB activation. To examine whether NF-κB activation events were inhibited by 5AZ, cells were stimulated with PGN with or without 5AZ for 30 min. Cytosolic IκBα degradation and nuclear translocation of p65, a subunit of NF-κB, were induced by PGN and were not influenced by 5AZ (Fig.3C).

To determine whether 5AZ inhibited iNOS promoter activity, iNOS promoter-luciferase reporter gene was transiently transfected to RAW264.7 cells. After stimulation with PGN for 30 min. or 1, 4, 8 or 24 hrs, iNOS promoter activity was assessed. Peptidoglycan increased iNOS promoter activity after 4 hrs and 5AZ inhibited PGN-induced iNOS promoter activity. Furthermore, iNOS promoter activity was increased by PGN in a time-dependent manner. 5-azacytidine inhibited PGN-induced iNOS promoter activity by 44.42% at 4 hrs and by 34.61% at 24 hrs (Fig.3D).

### Effect of 5AZ administration on LV function in a rat MI model

Two weeks after MI, LV function was evaluated by echocardiography and haemodynamic analysis (Table1). Heart rate did not differ significantly in all the groups. LV end-diastolic dimension and LVESD were smaller in the MI + 5AZ group than in MI group. EF (59.00 ± 8.03% *versus* 42.52 ± 2.58%), and FS (27.57 ± 5.14% *versus* 18.38 ±1.10%) were higher in MI + 5AZ group than in MI group. Haemodynamic changes were also improved by 5AZ administration. LV end-diastolic pressure was 5.38 ± 0.94 mmHg in the MI + 5AZ group, whereas 12.83 ± 1.85 mmHg in the MI group. Ventricular contractility (LV dP/dt-max, 8299.76 ± 411.56 mmHg *versus* 6610.36 ±282.37 mmHg) and relaxation indices (LV dP/dt-min, −4661.37 ±210.73 mmHg *versus* −4219.50 ± 162.98 mmHg) were improved after 5AZ administration.

**Table 1:** LV function in rat MI model. LV function was evaluated 2 weeks after MI

	Non-MI (5)	MI (*n* = 6)	MI+5AZ (*n* = 6)
HR (beats/min.)	306.24 ± 17.30	316.40 ± 24.21	314.04 ± 6. 89
LVEDD (mm)	6.52 ± 0.82	10.10 ± 0.23	7.35 ± 0.91*
LVESD (mm)	4.04 ± 0.81	8.12 ± 1.77	5.35 ± 0.77*
EF (%)	73.30 ± 8.13	42.52 ± 2.58	59.00 ± 8.03*
FS (%)	37.94 ± 6.92	18.38 ± 1.10	27.57 ± 5.14*
LVEDP (mmHg)	3.67 ± 0.95	12.83 ± 1.85	5.38 ± 0.94*
Max dp/dt	8523.08 ± 271.37	6610.36 ± 282.37	8299.76 ± 411.56*
Min dp/dt	-4716.07 ± 557.65	-4219.50 ± 163.98	-4661.37 ± 210.73*

LVEDD, LV end-diastolic dimension;

LVESD LV end-systolic dimension;

EF, ejection fraction;

FS, fractional shortening;

LVEDP, LV end-diastolic pressure;

MI, myocardial infarction.

* *P* < 0.05 *versus* MI.

### Effect of 5AZ on cardiac fibrosis and infiltrated macrophages in infarcted myocardium

**Figure 4:**
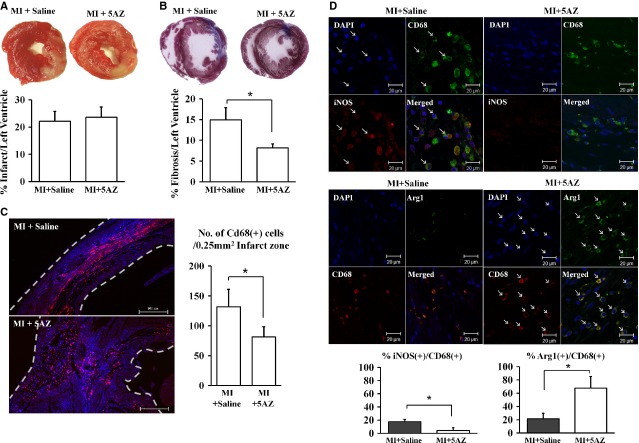
Effect of 5AZ administration on fibrosis and macrophage phenotypes in infarcted myocardium. (A) Myocardial infarction (MI) was induced and infarct size was measured after 1 day. There was no difference between the MI+saline group and the MI+5AZ group. (B) Cardiac fibrosis induced by myocardial infarction was reduced by 5AZ administration. Two weeks after myocardial infarction, fibrosis was evaluated by Masson's trichrome staining. Fibrotic changes were detected as blue colour and were quantified for expression as a graph. (C) Infiltrated CD68(+) macrophages in infarcted myocardium were counted and the cell numbers were expressed in a graph. (D) iNOS-expressing CD68(+) macrophages were observed in infarcted myocardium, but were significantly reduced by 5AZ administration (upper panels). Arginase-1 (Arg1)-expressing CD68(+) macrophages were predominant in 5AZ- administered infarcted myocardium (lower panels). The iNOS or Arg1-expressing CD68(+) macrophages were calculated and expressed as bar graphs. *P < 0.05 *versus* MI group; scale bar: 500 μm.

To investigate the role of 5AZ in MI, MI was induced by permanent ligation of the left anterior descending coronary artery in rats. To confirm whether the surgery was induced similar MI in all animals, infarct size was assessed at 24 hrs. Infarct size was similar in the two groups (Fig.4A). Then, 5AZ or saline was administered, and heart tissue was harvested 2 weeks after MI. Cardiac fibrosis was reduced to 8.14 ± 1.00% by 5AZ administration from 14.93 ± 2.98% (Fig.4B).

Immunohistochemical staining demonstrated that CD68(+) macrophages were infiltrated in the infarcted lesion after MI in both saline-administered and 5AZ-administered rats. The number of CD68(+) macrophages was 131.60 ± 29.52/0.25 mm^2^ in the MI group and 81.33 ± 17.06/0.25 mm^2^ in the MI + 5AZ group (*P* < 0.05, Fig.4C). To identify the subtype of infiltrated macrophages in infarcted myocardium, the expression of iNOS, an M1 macrophage marker, and Arg1, an M2 macrophage marker, was examined in infarcted myocardium (Fig.4D). Inducible nitric oxide synthase-expressing CD68(+) macrophages were observed in hearts from saline-administered rats after MI (17.75 ± 3.14 in the MI group *versus* 4.29 ± 4.34 in the MI + 5AZ group, *P* < 0.05), and Arg1-expressing CD68(+) macrophages were more frequent in 5AZ-administered heart (21.55 ± 8.06 in the MI group *versus* 67.85 ± 17.37 in the MI + 5AZ group, *P* < 0.05). Thus, 5AZ administration after MI increased the frequency of Arg1-expressing CD68(+) macrophages while decreased that of iNOS-expressing CD68(+) macrophages.

### Effect of 5AZ on angiotensin II-infused cardiac fibrosis

**Figure 5:**
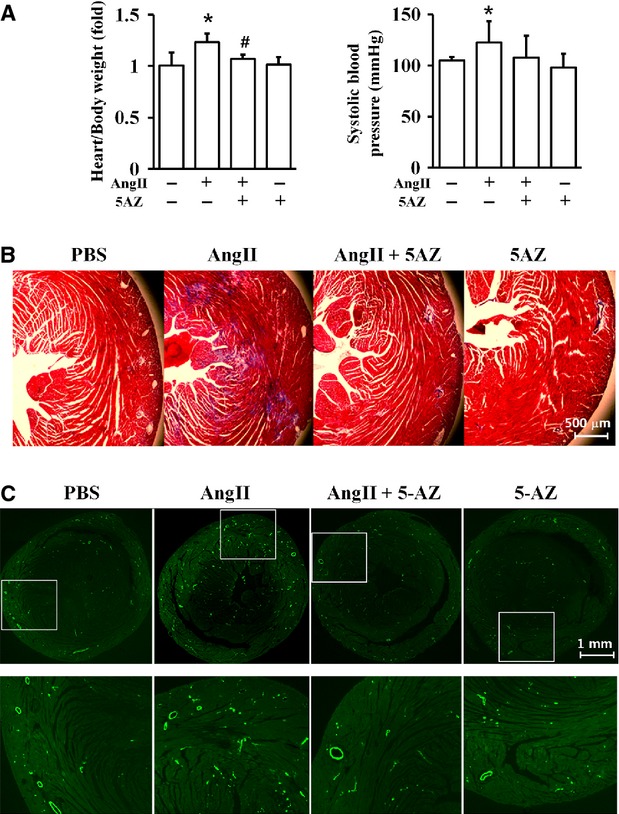
Effect of 5AZ administration on angiotensin II (AngII)-induced fibrosis. (A) AngII-induced cardiac hypertrophy was blocked by 5AZ administration in mice. (B) Blood pressure (mmHg) was measured after 2 weeks of AngII administration. (C) Cardiac fibrotic changes were assessed 2 weeks after implantation of AngII-releasing osmotic minipump with or without 5AZ administration. (D) Interstitial expression of α-smooth muscle actin was increased in AngII-stimulated heart, but was reduced by 5AZ administration.

To demonstrate the anti-fibrotic effect of 5AZ, we used AngII infusion in mice, another cardiac fibrosis model. AngII induced cardiac hypertrophy, which was attenuated after 5AZ administration (Fig.5A). On the other hand, 5AZ had no significant effect on systolic blood pressure in AngII-infused mice (Fig.5B). Cardiac fibrosis, mainly interstitial fibrosis, was significantly increased by AngII infusion, whereas it was reduced by 5AZ administration (Fig.5C). Expression of α-smooth muscle actin, a marker of activated myofibroblast, was increased by AngII infusion, and 5AZ administration reduced α-smooth muscle actin expression (Fig.5D). Unlike MI, AngII-infused myocardium seldom had infiltrated macrophages (data not shown).

**Figure 6:**
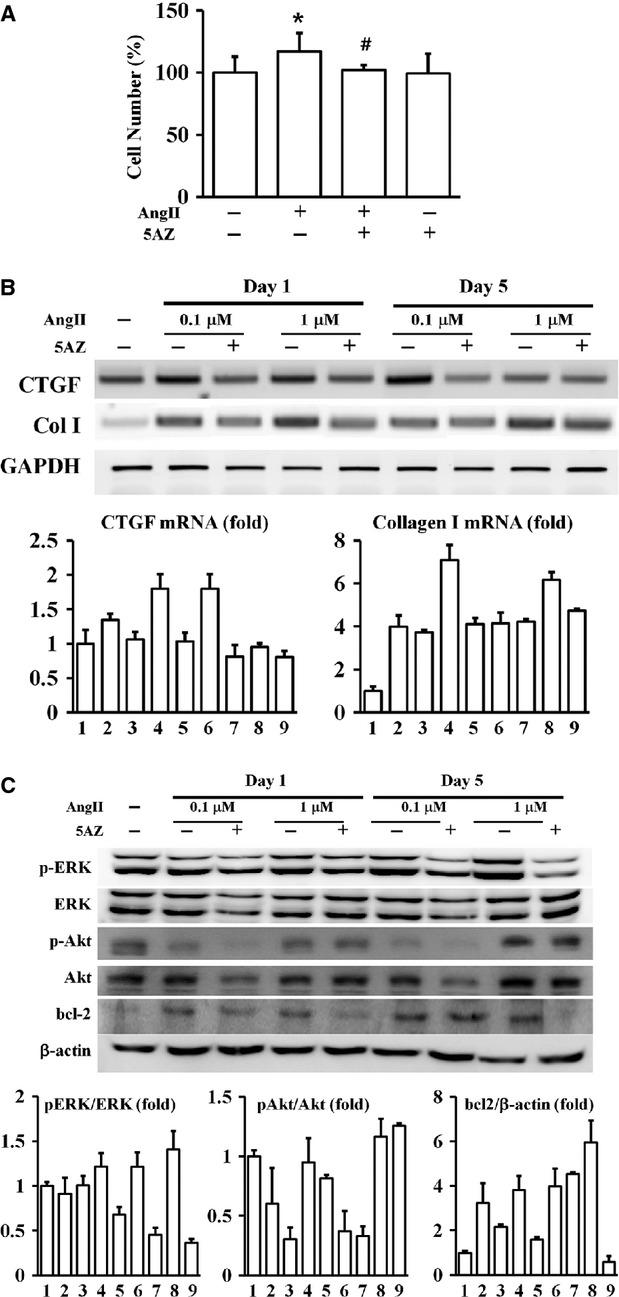
Effect of 5AZ on angiotensin II (AngII)-stimulated cardiac fibroblasts. (A) The cell number of cardiac fibroblasts was significantly increased after treatment with AngII for 5 days, and 5AZ blocked AngII-induced proliferation. (B) Increased mRNA of connective tissue growth factor (CTGF) and collagen type I (ColI), fibrosis-mediators, was reduced after 5AZ treatment. (C) AngII-induced expression of phosphorylated extracellular signal-regulated kinase, phosphorylated Akt and bcl-2 was reduced after 5AZ treatment. In bar graphs; (1) control; (2) AngII 0.1 μM for 1 day; (3) AngII 0.1 μM + 5AZ for 1 day; (4) AngII 1 μM for 1 day; (5) AngII 1 μM + 5AZ for 1 day; (6) AngII 0.1 μM for 5 days; (7) AngII 0.1 μM + 5AZ for 5 days; (8) AngII 1 μM for 5 days; (9) AngII 1 μM + 5AZ for 5 days.

To further evaluate the effect of 5AZ on cardiac fibrosis, fibroblasts were isolated and the cellular behaviour in response to a fibrotic stimulant, AngII, was examined to complement the *in vivo* findings. Because AngII was used to induce fibrosis *in vivo*, we focused on the effect of 5AZ on pro-fibrotic properties in AngII-stimulated cardiac fibroblasts. The cell number of cardiac fibroblasts was increased by AngII for 5 days (116.78 ± 14.85% in AngII-treated cells *versus* 100.00 ± 12.85% in PBS treated cell, *P* < 0.05), and the AngII-stimulated cell number was significantly reduced after 5AZ treatment (116.78 ± 14.85% in AngII-stimulated cells *versus* 102.05 ± 4.12% in AngII + 5AZ-treated cells, *P* < 0.05, Fig.6A).

Connective tissue growth factor and ColI are representative fibrotic mediators, and their mRNA expression was analysed in AngII-stimulated cardiac fibroblasts at day 1 and day 5. Connective tissue growth factor and ColI mRNA was induced by AngII stimulation with 0.1 or 1 μM and this was reduced after 5AZ treatment (Fig.6B). Extracellular signal-regulated kinase and Akt are pro-proliferative enzymes that are activated by phosphorylation, and bcl-2 is an anti-apoptotic protein. Phosphorylated ERK, phosphorylated Akt and bcl-2 were increased by AngII and attenuated by 5AZ (Fig.6C). These results suggested that 5AZ attenuated cell proliferation through reduction in bcl-2 induction as well as the activations of ERK and Akt in AngII-stimulated cardiac fibroblasts.

## Discussion

This study was designed to demonstrate whether the macrophage phenotype is modulated by 5AZ in myocardial infarction. We focused on two main events that occur in MI; activation of infiltrated macrophages and cardiac fibrosis.

Our study showed that 5AZ mediates a switch of macrophages to an anti-inflammatory phenotype that may be associated with recovery of LV function after MI. Furthermore, the anti-fibrotic effect of 5AZ is an additive beneficial action that contributes to promoting cardiac performance.

Inducible nitric oxide synthase is primarily induced in macrophages to participate in innate immunity and was selected here as a marker of M1 macrophages. Unlike eNOS and neural NOS, iNOS is synthesized *de novo* in response to various inflammatory stimuli. Induction of iNOS results in the production of large amounts of nitrite, and excessively produced nitric oxide causes tissue damage and contributes to pathology in a variety of inflammatory conditions. Inducible nitric oxide synthase in myocytes is protective and iNOS in peripheral blood cells is detrimental in during MI [12]. The effect of iNOS is divergent in myocytes and inflammatory cells, and selective iNOS deletion in peripheral blood cells limits infarct size in ischaemia-reperfusion injured mice [5,12].

The transcription factor NF-κB mediates the expression of iNOS and COX-2 in inflammatory responses. On stimulation with PGN, iNOS and COX-2 are induced in RAW264.7 macrophages. But 5AZ selectively inhibited iNOS expression without an effect on COX-2 expression. To address the mechanism of the inhibitory effect of 5AZ on iNOS, we examined the involvement of NF-κB in PGN-stimulated RAW264.7 macrophages. Degradation of IκBα and nuclear translocation of p65 were observed in PGN-stimulated RAW264.7 cells, however, 5AZ did not inhibit NF-κB activation. Although NF-κB appears to play an essential role in iNOS activation and expression, its participation in PGN-inducible activation is incompletely understood.

Next, we examined iNOS promoter activity. In a time-course promoter study, iNOS promoter activity began to increase in response to PGN at 4 hrs and this increase was sustained to 24 hrs. 5AZ consistently inhibited PGN-induced iNOS promoter activity, and this result was consistent with the results of nitric oxide production and iNOS expression.

Following tissue injury, macrophages usually exhibit an inflammatory M1 phenotype and release inflammatory mediators including nitric oxide, reactive oxygen intermediates, tumour necrosis factor-α (TNF-α) and interleukin-1 (IL-1). On the other hand, M2 macrophages exhibit anti-inflammatory activity and participate in wound healing.

Arg1-expressing macrophages suppress helper T cell2 (Th2)-dependent inflammation and fibrosis [13]. Macrophage-specific Arg1 exhibits both anti-inflammatory and anti-fibrotic activity in Th2-driven inflammation and fibrosis. In our rat MI model, 5AZ administration increased the frequency of macrophage-specific Arg1 expression and reduced iNOS-expressing macrophages in infarcted myocardium. The cardiac performance was significantly improved with the reduction in cardiac fibrosis by 5AZ administration after MI.

Cardiac fibrosis is a common outcome in cardiac damage of any cause, and it is associated with the activity of cardiac fibroblasts. Activated cardiac fibroblasts generate scarring within the tissue that ultimately leads to fibrosis. To focus on the effect of 5AZ on fibrosis, we used AngII-induced cardiac fibrosis. AngII infusion induced cardiac interstitial fibrosis and also significantly increased α-smooth muscle actin. 5AZ administration reduced both cardiac fibrosis and α-smooth muscle actin expression in AngII-infused mice. Cardiac fibroblasts proliferate and produce extracellular matrix in response to pathological stimuli and ultimately contribute to cardiac fibrosis. Extracellular signal-regulated kinase and Akt were reported to be activated in AngII-stimulated cardiac fibroblasts [14]. Bcl-2 is involved in the resistance of cardiac fibroblast to apoptosis and regulates cell survival [15]. We showed that 5AZ treatment to AngII-stimulated cardiac fibroblasts inhibited cell proliferation, ERK activation, Akt activation and bcl-2 induction.

Significant differences were found in the frequency of iNOS-expressing macrophages or Arg1-expressing macrophages in infarcted myocardium. The distribution of macrophages with the M1 or M2 phenotype is related to the repair process. Many reports have shown that phenotypic polarization of macrophages in the pathological environment is critical for both induction and resolution of the inflammatory response [2,15]. Uncontrolled pro-inflammatory activation may be deleterious to the host microenvironment thus resulting in delayed repair [8]. There is an association between M1 monocyte response and poor functional outcome after MI patients [16].

M1 and M2 macrophages can serve distinct functions in the regulation of the inflammatory response [17]. The molecular mechanisms that govern M1–M2 polarization remain incompletely understood. Several mechanisms that regulate macrophage polarization and plasticity have been demonstrated. Nuclear factor kappa B activation has been suggested to control the plasticity of macrophages in pathological conditions. In cancer-related inflammation, NF-κB activation in M1 macrophages is an essential event that contributes to tissue damage, neoplastic transformation and neoplastic cell proliferation [18]. Interferon response factors, signal transducer and activator of transcription, and activation protein 1 are central transcription factors that regulate the expression of pro-inflammatory cytokines related to M1-polarized macrophages [19,20]. Peroxisome proliferator-activated receptor γ has been shown to be an important determinant of M2 macrophages, [21] and Kruppel-like factor 4 has a demonstrated role in regulating M2 polarization in a myeloid-specific knockout model [22].

As 5AZ has been known to induce cardiac differentiation of bone marrow mesenchymal stem cells [9,10], further studies are needed to examine the possibility of cardiac regeneration and stem cell immobilization from bone marrow and to manage the potential side effects of 5AZ for clinical application.

**Figure 7:**
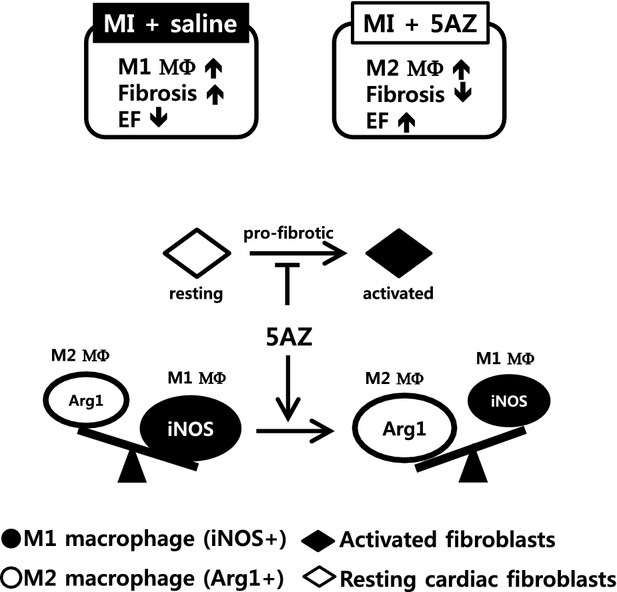
A proposed mechanism of 5AZ in the modulation of macrophage phenotype and cardiac fibroblasts.

Here, we have shown that 5AZ preferentially shifts macrophages to the M2 phenotype following MI and attenuates the pro-fibrotic behaviours of stimulated cardiac fibroblasts (Fig. 7). Thus, 5AZ can be utilized for diverse purposes and has potential as a promising therapeutic reagent.

## References

[1] Cleutjens JP, Blankesteijn WM, Daemen MJ (1999). The infarcted myocardium: simply dead tissue, or a lively target for therapeutic interventions. Cardiovasc Res.

[2] Gordon S, Taylor PR (2005). Monocyte and macrophage heterogeneity. Nat Rev Immunol.

[3] Lambert JM, Lopez EF, Lindsey ML (2008). Macrophage roles following myocardial infarction. Int J Cardiol.

[4] Dayan V, Yannarelli G, Billia F (2011). Mesenchymal stromal cells mediate a switch to alternatively activated monocytes/macrophages after acute myocardial infarction. Basic Res Cardiol.

[5] Troidl C, Mollmann H, Nef H (2009). Classically and alternatively activated macrophages contribute to tissue remodelling after myocardial infarction. J Cell Mol Med.

[6] Xie QW, Kashiwabara Y, Nathan C (1994). Role of transcription factor NF-kappa B/Rel in induction of nitric oxide synthase. J Biol Chem.

[7] Kim YS, Kwon JS, Cho YK (2012). Curcumin reduces the cardiac ischemia-reperfusion injury: involvement of the toll-like receptor 2 in cardiomyocytes. J Nutr Biochem.

[8] Taylor SM, Jones PA (1979). Multiple new phenotypes induced in 10T1/2 and 3T3 cells treated with 5-azacytidine. Cell.

[9] Makino S, Fukuda K, Miyoshi S (1999). Cardiomyocytes can be generated from marrow stromal cells *in vitro*. J Clin Invest.

[10] Mohanty S, Bose S, Jain KG (2013). TGFbeta1 contributes to cardiomyogenic-like differentiation of human bone marrow mesenchymal stem cells. Int J Cardiol.

[11] Bechtel W, McGoohan S, Zeisberg EM (2010). Methylation determines fibroblast activation and fibrogenesis in the kidney. Nat Med.

[12] Guo Y, Sanganalmath SK, Wu W (2012). Identification of inducible nitric oxide synthase in peripheral blood cells as a mediator of myocardial ischemia/reperfusion injury. Basic Res Cardiol.

[13] Pesce JT, Ramalingam TR, Mentink-Kane MM (2009). Arginase-1-expressing macrophages suppress Th2 cytokine-driven inflammation and fibrosis. PLoS Pathog.

[14] Olson ER, Naugle JE, Zhang X (2005). Inhibition of cardiac fibroblast proliferation and myofibroblast differentiation by resveratrol. Am J Physiol Heart Circ Physiol.

[15] Mayorga M, Bahi N, Ballester M (2004). Bcl-2 is a key factor for cardiac fibroblast resistance to programmed cell death. J Biol Chem.

[16] van der Laan AM, Hirsch A, Robbers LF (2012). A proinflammatory monocyte response is associated with myocardial injury and impaired functional outcome in patients with ST-segment elevation myocardial infarction: monocytes and myocardial infarction. Am Heart J.

[17] Mosser DM, Edwards JP (2008). Exploring the full spectrum of macrophage activation. Nat Rev Immunol.

[18] Biswas SK, Sica A, Lewis CE (2008). Plasticity of macrophage function during tumor progression: regulation by distinct molecular mechanisms. J Immunol.

[19] Colonna M (2007). TLR pathways and IFN-regulatory factors: to each its own. Eur J Immunol.

[20] Vakkila J, Demarco RA, Lotze MT (2008). Coordinate NF-kappaB and STAT1 activation promotes development of myeloid type 1 dendritic cells. Scand J Immunol.

[21] Odegaard JI, Chawla A (2011). Alternative macrophage activation and metabolism. Annu Rev Pathol.

[22] Liao X, Sharma N, Kapadia F (2011). Kruppel-like factor 4 regulates macrophage polarization. J Clin Invest.

